# Ferritin and Liver Steatosis in Children: Interactions Between Metabolic Clustering and *PNPLA3* Variants

**DOI:** 10.3390/ijms27073044

**Published:** 2026-03-27

**Authors:** Mihaela-Andreea Podeanu, Raluca Elena Sandu, Bianca Ștefănița Vintilescu, Claudiu Marinel Ionele, Ion Rogoveanu, Ioana Streață, Carmen Elena Niculescu, Dan Nicolae Florescu, Sergiu-Marian Cazacu, Ștefania Cornelia Andrei, Adina Maria Barbu, Mioara Desdemona Stepan

**Affiliations:** 1 Doctoral School, University of Medicine and Pharmacy of Craiova, 200349 Craiova, Romania; mihaela.podeanu@umfcv.ro; 2 Department of Infant Care, Pediatrics and Neonatology, University of Medicine and Pharmacy of Craiova, 200349 Craiova, Romania; bianca.vintilescu@umfcv.ro (B.Ș.V.);; 3 Department of Biochemistry, University of Medicine and Pharmacy of Craiova, 200349 Craiova, Romania; 4 Department of Gastroenterology, University of Medicine and Pharmacy of Craiova, 200349 Craiova, Romania; claudiu.ionele@umfcv.ro (C.M.I.); sergiu.cazacu@umfcv.ro (S.-M.C.); 5 Human Genomics Laboratory, University of Medicine and Pharmacy of Craiova, 200349 Craiova, Romania; ioana.streata@umfcv.ro (I.S.);

**Keywords:** hepatic steatosis, *PNPLA3*, metabolic risk, ferritin, children

## Abstract

Metabolic abnormalities are frequently associated with hepatic steatosis and low-grade inflammation, yet the contributions of iron metabolism and genetic susceptibility are not fully understood. We aimed to investigate the relationship between serum ferritin, hepatic steatosis, metabolic risk clustering, and the *PNPLA3* rs738409 gene variant in children. A total of 68 children aged 6–14 years underwent anthropometric, biochemical, imaging, and genetic assessment. Hepatic steatosis was present in 72.1% of participants, with fibrosis greater than F1 in 42.6%. Serum ferritin showed a strong correlation with echographic liver steatosis severity (ρ = 0.804, *p* < 0.001) and a moderate correlation with the number of metabolic risk components (ρ = 0.482, *p* < 0.001). The highest metabolic burden occurred in children with low iron and elevated ferritin. While *PNPLA3* status did not independently predict ferritin levels, carriers had a significantly higher prevalence of hypertension (50.0% vs. 25.0%, *p* = 0.038) and a non-significant trend toward low HDL-C (65.0% vs. 42.9%, *p* = 0.070). Ferritin was associated with metabolic clustering and ultrasound-defined hepatic steatosis, acting as a nonspecific marker of combined metabolic and hepatic alterations. *PNPLA3* genotype was not independently related to ferritin or fibrosis in early pediatric disease. Given the cross-sectional design and the relatively small sample size, these findings should be interpreted as exploratory and further studies including larger populations and direct inflammatory markers should be conducted.

## 1. Introduction

Weight gain in childhood imposes a substantial and growing burden on healthcare systems [1]. This burden is sustained by unfavorable dietary patterns characterized by high consumption of sugars, fast foods, and refined grains, alongside insufficient intake of fruits and vegetables. Evidence consistently shows that dietary improvements can reduce body mass index (BMI) and enhance long-term health outcomes [2]. However, diet alone does not fully explain the rise in pediatric obesity and the increasing number of children requiring specialized care [3].

In the pediatric population, the most recognized complications of obesity are metabolic syndrome (MetS), Metabolic Dysfunction-Associated Steatotic Liver Disease (MASLD)—previously known as Metabolic Dysfunction-Associated Fatty Liver Disease (MAFLD) and before that, Non-Alcoholic Fatty Liver Disease (NAFLD)—and type 2 Diabetes Mellitus (T2DM) [4]. The true implications of these conditions lie in their early onset during childhood, as they negatively affect quality of life and are associated with early complications. Moreover, their progressive nature means that many affected children transition into adulthood with persistent disease, requiring increasing levels of medical care over time [5].

When considering MetS and MASLD, the contribution of obesity to the initiation and progression of these conditions is well established [6,7]. However, dietary factors alone do not explain this transition. Multiple additional factors contribute to the progression from excess adiposity to metabolic and hepatic dysfunction. These include genetic susceptibility, such as *Patatin-like phospholipase domain-containing protein 3* (*PNPLA3*) variants in MASLD, chronic low-grade inflammation, alterations in insulin sensitivity, dysregulation of lipid metabolism, gut microbiota, physical inactivity, environmental and socioeconomic influences and many others [8].

Among the multitude of factors involved in the progression from obesity to metabolic and hepatic dysfunction, our attention in the present study was directed toward chronic low-grade inflammation and genetic susceptibility. Serum ferritin was selected as a biomarker potentially reflecting metabolic and inflammatory processes, although it represents a nonspecific marker while genetic susceptibility was assessed through the evaluation of *PNPLA3* variants.

We selected serum ferritin for assessing inflammation due to its availability and familiarity for clinicians. Beyond its well-known role in storing iron, it behaves as an acute-phase reactant [9]. It is a widely available laboratory parameter, routinely measured in clinical practice. Furthermore, its testing is standardized, cost-effective, and accessible even in resource-limited settings, making it a pragmatic biomarker for pediatric research [10]. Numerous studies on both adult and pediatric populations have linked elevated ferritin levels to MASLD and MetS, indicating connections with lipid abnormalities, and insulin resistance, proposing it as a desirable marker [11,12,13].

A single-nucleotide variant of the *PNPLA3* gene, which is found in the liver and adipose tissue, is associated with obesity and hepatic fat accumulation, in both adults and children [14]. The rs738409 polymorphism, determining the I148M substitution, has, by itself, been linked to higher prevalence and the severity of hepatic steatosis and progression to steatohepatitis to the point of fibrosis [15]. A study on a cohort of children with Down syndrome showed that the *PNPLA3* polymorphisms are associated with the increase in interleukin-6 [16]—a sensitive, fast acting proinflammatory cytokine [17].

Despite the increasing recognition of MASLD in children, the interactions between metabolic dysfunction, inflammatory processes, iron metabolism, and genetic susceptibility remain incompletely understood. Serum ferritin has been proposed as a potential marker reflecting both metabolic and hepatic alterations, but its relationship with metabolic risk clustering and genetic determinants such as the *PNPLA3* rs738409 variant in pediatric populations has not been fully clarified.

To our knowledge, few pediatric studies have simultaneously evaluated ferritin, metabolic risk clustering, hepatic ultrasound findings, and *PNPLA3* genetic variability. The aim of the present study was to investigate the associations between serum ferritin levels, ultrasound assessed hepatic steatosis, metabolic risk clustering (derived from MetS criteria), and the *PNPLA3* rs738409 polymorphism in a cohort of children aged 6–14 years.

## 2. Results

A total of 68 children were included in the study, with a median age of 10.5 years (8.0–12.0). The study population was predominantly male (50 boys, 73.5%), and slightly more than half of the participants were residents in an urban environment (54.4%).

Regarding anthropometric parameters, children exhibited a median BMI of 25.65 kg/m^2^ (19.1–29.3), with 70.6% presenting a waist circumference at or above the 90th percentile according to age and sex indices. The median waist-to-height ratio (WHtR) was 0.59 (0.54–0.64). Elevated blood pressure values were observed in 39.7% of subjects.

The mean fasting glycemia was 83.04 ± 11.93 mg/dL, with 5.9% of children presenting elevated fasting glucose levels. Dyslipidemia was frequent, with 45.6% of participants exhibiting elevated triglycerides and 55.9% presenting low high-density lipoprotein cholesterol (HDL-C) values. The clustering of metabolic abnormalities was reflected by a median of 3 metabolic risk components adapted for age.

Markers of iron metabolism showed a median serum iron level of 11.0 µg/dL (8.0–18.0) and a median serum ferritin concentration of 191.5 ng/mL (92.3–213.8). The assessment showed a predominance of the low serum iron/elevated ferritin pattern, observed in 50 participants (73.5%). A completely normal profile, with both parameters within reference ranges, was identified in only 15 patients (22.1%).

Hepatic involvement was common, with 72.1% of children presenting ultrasound evidence of fatty liver disease. Approximately 60% of the participants presented moderate to severe echographic scores. Fibrosis assessment showed a cumulative 42.6% of children had F1 or F2 fibrosis, with a median value of 5.7 kPa (3.5–7.05). Importantly, no participant presented fibrosis greater than stage F2.

Regarding genetic characteristics, 59.0% of the study population carried at least one copy of the *PNPLA3* risk allele, with genotype frequencies of 41.2% C/C, 42.6% C/G, and 16.2% G/G, respectively.

More information regarding the descriptives of the group can be found in Table 1.

*PNPLA3* carrier status was not associated with elevated fasting glycemia, having a low prevalence in both groups (3.6% in non-carriers vs. 7.5% in carriers; Fisher’s exact *p* = 0.638). In contrast, carriers presented a significantly higher prevalence of high triglycerides compared with non-carriers (57.5% vs. 28.6%; χ^2^ = 5.557, *p* = 0.018; Fisher’s exact *p* = 0.026). A higher frequency of low HDL-C was observed among carriers (65.0% vs. 42.9%), but without being statistically significant (χ^2^ = 3.276, *p* = 0.070; Fisher’s exact *p* = 0.086). Moreover, *PNPLA3* carrier status was significantly associated with elevated blood pressure, with hypertension present in 50.0% of carriers compared with 25.0% of non-carriers (χ^2^ = 4.300, *p* = 0.038; Fisher’s exact *p* = 0.047).

Continuous metabolic parameters were further compared according to *PNPLA3* carrier status using the Mann–Whitney U test. Children carrying the *PNPLA3* risk allele presented significantly higher waist circumference (U = 348.0, *p* = 0.008), SBP (U = 384.5, *p* = 0.029), DBP (U = 319.5, *p* = 0.003), and triglycerides (U = 326.0, *p* = 0.003) compared with non-carriers. However, no statistically significant differences were observed for WHtR (*p* = 0.186), fasting glycemia (*p* = 0.112), or HDL-C (*p* = 0.363).

Echographic severity tended to be higher in children carrying the *PNPLA3* risk allele compared to non-carriers; however, this difference did not reach statistical significance (Mann–Whitney U test, *p* = 0.393). *PNPLA3* carriers showed a higher mean rank echographic score compared to non-carriers (36.14 vs. 32.16).

*PNPLA3* carrier status was not significantly associated with fibrosis grades. Although carriers showed a higher mean rank fibrosis score compared with non-carriers (36.83 vs. 31.18), the difference did not reach statistical significance (Mann–Whitney U = 467, *p* = 0.192). When fibrosis severity was analyzed according to *PNPLA3* genotype, a progressive increase in liver stiffness was observed from C/C to C/G and G/G genotypes (mean rank: 29.95, 34.98, and 44.82, respectively). However, this trend did not reach statistical significance (Kruskal–Wallis H = 4.506, *p* = 0.105).

Significant differences in echographic severity were observed across the four iron–ferritin classifications (Kruskal–Wallis test, χ^2^ = 36.666, *p* < 0.001), with the highest scores in children presenting low serum iron and elevated ferritin levels. A similar pattern was observed for metabolic risk clustering, as the number of metabolic components also differed significantly between iron–ferritin phenotypes (χ^2^ = 20.740, *p* < 0.001), the greatest metabolic burden being observed in the low-iron/high-ferritin group. No statistically significant differences in *PNPLA3* carrier status were detected across these categories, although a trend was noted (*p* = 0.067).

Serum ferritin showed a moderate positive correlation with the number of metabolic risk components (Spearman’s ρ = 0.482, *p* < 0.001). It also positively correlated with triglyceride levels (ρ = 0.288, *p* = 0.017) and inversely with HDL-C (ρ = −0.443, *p* < 0.001). Positive correlations were also observed with waist circumference (ρ = 0.416, *p* < 0.001), WHtR (ρ = 0.432, *p* < 0.001), and fasting glycemia (ρ = 0.427, *p* < 0.001). Associations with blood pressure were weaker but remained significant for both systolic (ρ = 0.291, *p* = 0.016) and diastolic blood pressure (ρ = 0.457, *p* < 0.001).

A strong positive correlation was identified between ferritin and echographic score severity (ρ = 0.804, *p* < 0.001), which was stronger than the correlation between ferritin and metabolic risk clustering. Echographic severity also correlated strongly with liver stiffness measurements (ρ = 0.834, *p* < 0.001) and fibrosis grade (ρ = 0.753, *p* < 0.001), supporting the consistency of ultrasonographic assessment.

In multivariable linear regression analysis, with log-transformed ferritin as the dependent variable, echographic severity (B = 0.197, β = 0.310, *p* < 0.001), number of metabolic risk components (B = 0.078, β = 0.174, *p* = 0.002), and iron deficiency status (B = 1.023, β = 0.603, *p* < 0.001) were associated with ferritin levels in this exploratory model, whereas *PNPLA3* carrier status was not (*p* = 0.412). The model was statistically significant (F = 115.020, *p* < 0.001) and explained 88.0% of ferritin variability (adjusted R^2^ = 0.872). Given the relatively small sample size and potential model overfitting, the regression analysis is interpreted as exploratory and aimed at identifying associations rather than establishing causal relationships.

**Figure 1:**
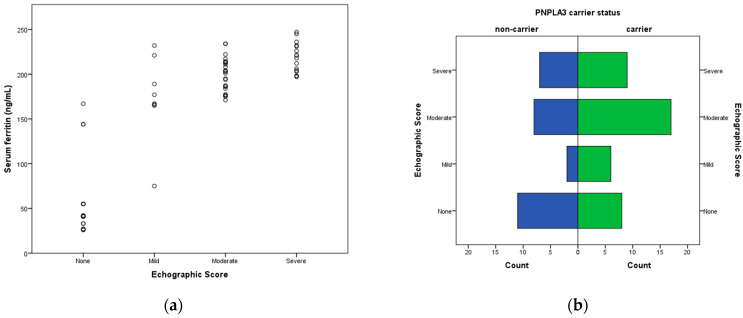
(**a**) Scatter plot illustrating the association between echographic score and log-transformed serum ferritin levels. A strong positive correlation was observed, indicating increasing ferritin levels with greater hepatic involvement (Spearman’s ρ = 0.804, *p* < 0.001). (**b**) Distribution of echographic severity categories (none, mild, moderate, severe) among *PNPLA3* non-carriers and carriers. *PNPLA3* carriers tended to present higher echographic severity, although between-group differences were not statistically significant.

The relationship between hepatic involvement and ferritin levels, as well as the distribution of liver disease severity according to genetic background, are illustrated in Figure 1.

## 3. Discussion

In the present study, metabolic risk clustering was defined using a composite approach rather than applying a strict diagnosis of MetS. This decision was based on the absence of a universally accepted international definition for pediatric MetS as most of the definitions are validated primarily for children aged 10 years and older. However, metabolic alterations consistent with MetS can be observed even in younger age groups, as highlighted by multiple researchers interested in this topic. To address this impairment, we partially adopted the criteria proposed by Zong et al. in 2024, which offers updated and clinically applicable thresholds for pediatric populations [18]. However, we did not fully adhere to their proposal to replace waist circumference with WHtR, as, in our opinion, and based on current data, it is yet to be validated as a diagnostic tool and requires further confirmatory studies [19]. Therefore, waist circumference percentiles remained the marker for central adiposity in our analysis. In this context, we also want to point out the need for a consensus regarding the definition for pediatric MetS and the need to lower the age of diagnosis.

Consistent with our results, multiple epidemiological studies have shown that abdominal obesity and dyslipidemia, characterized by high triglycerides and low HDL-C, are the most encountered metabolic abnormalities in children with obesity, MetS, and hepatic steatosis [20,21]. Insulin resistance and elevated blood pressure, the other metabolic risk components we searched for, are less frequently reported [22]. Several longitudinal studies reported that there is a temporal sequence of cardiometabolic components, with adiposity being the first step, followed by insulin resistance, and then hypertension. Also, children presenting cardiovascular risk clustering before and during puberty will become adults with an increased cardiovascular risk [23].

The prevalence of hepatic steatosis and early-stage fibrosis reported in our cohort partially aligns with pediatric data, reinforcing the fact that fatty-liver disease is already widespread among children with metabolic disturbances. A recent meta-analysis, including 176 studies with a total of 57,058 children, determined the global prevalence of MASLD/MAFLD in children with excess weight is approximately 41%. Prevalence varied across countries and was higher in hospital-based cohorts [24], which may explain the fact that in our cohort we observed a high frequency of ultrasound-evidenced hepatic steatosis (72.1%). However, this high percent can also be due to selection biases, as also stated in the section dedicated to study limitations.

There are studies suggesting that mild to moderate steatosis and fibrosis are present in a great proportion of children with overweight and obesity, whereas severe forms of fibrosis are rarely encountered [25]. However, the reported prevalence varies considerably across studies, mostly because no well standardized pediatric thresholds exist. Consequently, the use of different elastography cut-offs can substantially modify fibrosis estimates, with values ranging from low to nearly 50% among children with obesity [26]. Several studies have shown correlations between fibrosis scores, the increase in hepatic transaminases and other derivate scores, and excess fat accumulation [27]. Our group presented a significant percentage of F1 and F2 fibrosis (42.6%), without identifying severe cases (above F2). These results can be explained by the inclusion of children under 10 years of age, in whom disease progression is still limited. However, it is important to emphasize that, even at these young ages, we identified changes that can evolve over time, suggesting that liver involvement is common even in the youngest, but is predominant in early and potentially reversible stages.

Among the factors predisposing to fat accumulation in the liver, the *PNPLA3* I148M variant (rs738409) is currently considered one of the most important genetic determinants, independent of the degree of obesity or metabolic alterations [28]. It is responsible for the creation of a protein called adiponutrin, an enzyme predominantly expressed in hepatocytes and involved in lipid metabolism. It participates in the remodeling of intracellular lipid droplets and in triglyceride turnover. This facilitates the breakdown of triglycerides into fatty acids and glycerol so they can be further processed and utilized by the body. Through this role, hepatic lipid balance is maintained; alterations in its activity may lead to lipid accumulation in the liver and subsequent metabolic disturbances. The mutation is also responsible for enhancing lipogenesis and triggering inflammation and oxidative stress associated damages [29]. In our cohort, 59% of children carried at least one copy of the *PNPLA3* risk allele. This relatively high prevalence places our population within the range reported in pediatric studies and supports the concept that genetic predisposition is frequent among children evaluated for metabolic and hepatic abnormalities. However, the carrier frequency in our sample was slightly lower than that typically described in steatosis-selected cohorts.

Data regarding the rs738409 (G) allele of the *PNPLA3* gene show that approximately 60–85% of children with ultrasound-confirmed fatty liver carry at least one risk allele. In contrast, only about 23–41% of children without steatosis are carriers. This marked difference highlights the important role of genetic susceptibility in early hepatic fat accumulation. Moreover, a substantial proportion of affected children are homozygous for the variant, supporting a dose-dependent effect of the allele on liver fat content. Altogether, these observations indicate that *PNPLA3* is considered an important genetic factor associated with pediatric MASLD susceptibility, although its role may vary depending on the studied population. It contributes to disease development independently of traditional metabolic risk factors [30]. The G allele increases susceptibility for steatosis, but it does not promote insulin resistance or have an inhibitory effect on lipolysis. Instead, carriers had smaller adipocytes, suggesting that the genetic variant influences the development and function of adipose tissue, which can predispose to fat deposit build-up in the liver [31]. The G-allele confers susceptibility to extrahepatic complications, including early renal damage, even in the absence of biochemical liver injury [32,33].

The involvement of *PNPLA3* in the metabolic profile is inconclusive. Some studies have reported associations with metabolic changes, such as decreased HDL-C or alterations in glucose homeostasis or insulin resistance, while others have not identified significant differences in these parameters between carriers and non-carriers of the risk allele [34,35]. In our cohort, carriers exhibited a significantly higher prevalence of hypertriglyceridemia and elevated blood pressure, higher waist circumference and triglyceride levels, while fasting glycemia and HDL-C did not differ significantly between groups. These results suggest that the effects of *PNPLA3* on systemic metabolism are variable and likely dependent on the metabolic context and characteristics of the studied population.

Evidence suggests that the *PNPLA3* I148M variant is associated with severe histological liver injury, including steatohepatitis and fibrosis. This association has been observed across multiple adult and pediatric cohorts and supported by meta-analytic data, suggesting a potential role of the risk allele in disease progression rather than simple fat storage [36]. However, findings in pediatric populations are not entirely consistent. Some studies have failed to demonstrate a clear relationship between the *PNPLA3* locus and histological severity of NAFLD, whereas others reported a dose-dependent effect, with heterozygous and homozygous carriers exhibiting higher grades of fibrosis, stating that *PNPLA3* acts as a disease modifier rather than a uniform determinant of progression [37,38]. In our cohort, *PNPLA3* carrier status was not significantly associated with fibrosis grade, although carriers tended to show higher fibrosis values than non-carriers. When analyzed by genotype, liver stiffness gradually increased from C/C to C/G and G/G genotypes, but the difference did not reach statistical significance. These findings may indicate that, in children, the influence of *PNPLA3* on fibrogenesis may already be present but remains subtle, probably reflecting the early stage of disease and the shorter duration of metabolic exposure. However, given the relatively small sample size and the limited number of participants in each subgroup, particularly for the G/G genotype, these findings should be considered exploratory.

In a review published in 2022, Astarini et al. [39] confirmed the *PNPLA3* rs738409 G allele has been associated with higher serum ferritin levels in patients with NAFLD and with greater inflammatory infiltrates, by increasing the production of pro-inflammatory and pro-fibrinogenic cytokines. Through this mechanism, it promotes hepatic inflammation and contributes to fibrosis progression [39]. Alterations in iron metabolism in metabolic conditions may influence ferritin levels, making it difficult to distinguish between iron storage and metabolic-related changes [40]. Overall, *PNPLA3* polymorphisms appear to facilitate NAFLD progression—from steatosis to inflammation and fibrosis—partly through inflammation-driven mechanisms in which ferritin may act as a marker associated with disease severity [39].

In a previous study, we investigated ferritin’s link to iron metabolism and inflammation in the context of pediatric MetS and hepatic fat accumulation. Beyond its role as an intracellular iron storage protein, its role as an acute-phase reactant determined a reduced intestinal iron absorption, leading to changes in iron distribution and metabolism, potentially leading to functional iron deficiency despite adequate iron stores [41]. The predominance of low serum iron associated with elevated ferritin observed in our cohort may be consistent with alterations in iron metabolism described in metabolic and inflammatory conditions. A relatively new term that encompasses this concept is metaflammation—a chronic low-grade inflammatory state arising from excess nutrient intake and adipose tissue expansion, linking metabolic dysregulation to immune system activation [42].

In our cohort, the low iron/high ferritin phenotype was associated with more pronounced metabolic alterations, as well as higher hepatic ultrasound severity scores and fibrosis grades; this pattern may reflect an association between metabolic alterations and hepatic involvement rather than a defined causal pathway. However, in the absence of direct inflammatory markers, these interpretations remain speculative.

In children with obesity-related metabolic syndrome, persistent activation of innate immune pathways and increased secretion of pro-inflammatory cytokines generate a systemic inflammatory state that may contribute to insulin resistance, dyslipidemia, and hepatic steatosis [43]. Within this inflammatory milieu, ferritin levels rise with the number of metabolic syndrome components. They also correlate with insulin resistance, lipid abnormalities, abdominal adiposity [11,44,45,46], steatosis severity on ultrasound, and elevated liver enzymes [13]. Accordingly, ferritin may be associated with children presenting more advanced metabolic and hepatic alterations, although its role as a predictive marker remains uncertain [47]. Moreover, serum ferritin has been proposed as a non-invasive marker of fibrosis severity in pediatric NAFLD, with studies showing increased levels in parallel with elastographic and histologic fibrosis progression [48,49], findings that are consistent with those observed in our cohort.

The multivariable analysis identified associations between serum ferritin levels, hepatic ultrasound severity, the number of metabolic risk components, and iron deficiency status. These findings, which are exploratory and hypothesis-generating rather than indicative of robust independent relationships, suggest that ferritin may reflect the combined burden of metabolic alterations and liver involvement in this cohort. However, given the relatively small sample size and the cross-sectional design, these associations should be interpreted with caution as they are not establishing a causal relationship. In addition, the coexistence of metabolic and hepatic alterations supports its role as a nonspecific marker rather than a specific mechanistic indicator.

In the present cohort, ferritin levels were associated with both metabolic risk clustering and ultrasound-defined hepatic steatosis, while *PNPLA3* carrier status showed limited associations with ferritin levels and liver fibrosis. These findings may indicate that metabolic alterations and liver involvement are closely related to ferritin variability in this pediatric population. However, the current data do not allow conclusions regarding the relative contribution of metabolic, inflammatory, or genetic mechanisms to disease development. Given the cross-sectional design and the relatively small number of participants, particularly within the *PNPLA3* genotype subgroups, the present findings are exploratory and require confirmation in larger prospective studies.

This study has several limitations that should be acknowledged. First, the relatively small sample size has limited the statistical power. In addition, the single-center design and the inclusion of only participants with complete datasets may introduce selection bias. Although liver stiffness was assessed by transient elastography, the controlled attenuation parameter module was not available; therefore, hepatic steatosis was evaluated only by ultrasonography, which is operator-dependent and less sensitive for mild fat accumulation, particularly in pediatric populations. We were not able to evaluate the temporal relationships or disease progression, and no longitudinal follow-up was performed. The analyzed cohort had a high prevalence of hepatic steatosis, which can be due to selection bias, which limits the generalizability of the findings to the broader pediatric population. Furthermore, the fibrosis cutoffs used for elastography are not universally validated in pediatric populations, which may introduce a risk of fibrosis misclassification. The relatively young age of the cohort may reflect early disease stages. Additionally, the absence of direct inflammatory markers such as C reactive protein, interleukin-6, or hepcidin limits the ability to further characterize the inflammatory component and prevents mechanistic interpretation of ferritin variability. Future prospective longitudinal studies including larger pediatric populations, complete biochemical profiling, and advanced steatosis quantification are warranted to clarify the prognostic role of ferritin and its interaction with *PNPLA3* variants.

## 4. Materials and Methods

### 4.1. General Data About the Study

The present study was conducted across multiple levels and represents a secondary analysis derived from a broader prospective research project. The initial cohort included 100 pediatric patients enrolled in the study entitled “Utility of antioxidants in the prevention, monitoring and treatment of non-alcoholic fatty liver disease in children with established *PNPLA3* genotype”. The subjects were children and adolescents aged 6 to 14 years of age, and the selection period was between September 2021 and April 2022, being performed within the University of Medicine and Pharmacy Craiova. The participants were selected among the patients of the Pediatric I Department of the Emergency Clinical County Hospital of Craiova, Romania.

For the current study, the selection criteria were designed to match the aim of the study. First, all the participants needed to have the informed consent form signed by a legal representative to participate in the study, which in all cases was one of the parents (mostly the mother of the child).

**Figure 2:**
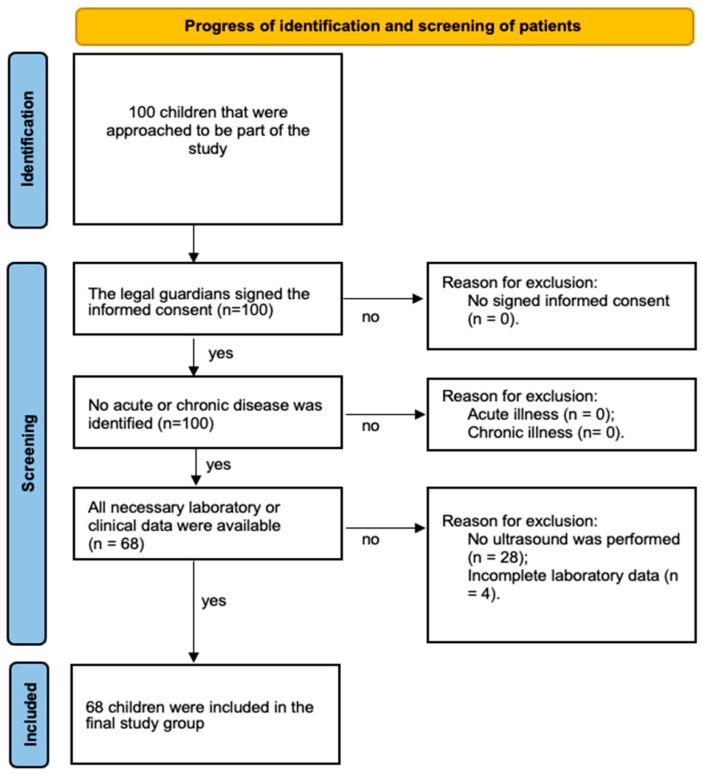
Flow diagram illustrating the selection of the study population.

Of the 100 initially approached children and adolescents, 68 were included in the present analysis. These participants had complete anthropometric, biochemical, imaging, and genetic data required for the variables investigated. Participants with incomplete laboratory or imaging data relevant to the present analysis were excluded. A visual representation of the selection process is presented in Figure 2.

Since the beginning, we excluded from the study any children and adolescents who showed signs of acute or chronic illness during anamnesis or clinical examination (current or within the three months prior to the examination) or who took iron supplements as it interfered with iron metabolism. The final requirement was that, at the end of the study, every participant included in the group for this particular study had to have all the investigations necessary for our analysis.

### 4.2. Patient Assessment

All participants were evaluated by a multidisciplinary pediatric team consisting of a senior pediatrician and a pediatric resident. The initial assessment included a structured interview with the parents or legal guardians, during which the purpose of the study and the procedures to be performed were clearly explained. Written informed consent was obtained from the parents or legal guardians prior to inclusion in the study.

Following consent, a detailed medical history was obtained, including information on the child’s personal medical history, family health status, and environmental and living conditions.

A comprehensive and meticulous clinical examination was systematically conducted for each participant, ensuring a complete evaluation of all major organ systems.

In the morning, anthropometric measurements were performed using standardized methods, as follows.

A thaliometer calibrated in 1 mm increments was utilized for measuring height, while a high-precision electronic scale was employed for weight measurement.

BMI was calculated as weight (kg) divided by height squared (m^2^) and participants were classified according to World Health Organization age- and sex-specific reference standards: normal weight (5th–<85th percentile), overweight (≥85th–<95th percentile), or obesity (≥95th percentile).

Waist circumference was measured at the end of expiration at the umbilical level using a dedicated measuring device. Values were interpreted using age- and sex-specific percentiles in accordance with the recommendations of the Childhood Obesity Task Force of the European Association for the Study of Obesity for the evaluation of overweight and obese children, based on which, a waist circumference at or above the 90th percentile is considered to increase the metabolic risk [50], being also used in most definitions of MetS in the pediatric population.

Blood pressure was measured in the morning, after a rest period of approximately ten minutes, with an electronic tensiometer. The calculation of percentiles for blood pressure values was performed according to age, gender, and height using an online software which is based on Centers for Disease Control and Prevention available data [51,52]. We considered it to be abnormal (high blood pressure), if the result was above the 90th percentile.

For liver examination, an abdominal ultrasound was performed by an experienced physician, using an adapted transducer, on a Hitachi Aloka Arietta 70 Diagnostic Ultrasound System (Hitachi Aloka Medical, Tokyo, Japan). Also, the same physician performed liver elastography, using ECHOSENS FibroScan 430 Mini with SN: F90497 (Echosens, Paris, France) Liver stiffness measurements were performed with the probe placed in the 7th or 8th intercostal space along the right anterior axillary line. The cut-off thresholds and definitions used in this study are detailed in the corresponding section.

A venous blood sample (6 mL) was obtained from each participant in the morning, after a prior fasting of at least 8 h (overnight), by a qualified nurse. Serum iron was measured colorimetrically and ferritin was quantified by electrochemiluminescence immunoassay (ECLIA). HDL-C, triglycerides, and fasting blood glucose were determined using standard laboratory methods routinely applied in clinical practice.

Genomic DNA was extracted from peripheral blood leukocytes obtained from all study participants using the Wizard^®^ Genomic DNA Purification Kit (Promega, Madison, WI, USA). Genotyping of the *PNPLA3*: c.444C>G (rs738409) variant was carried out for all subjects in the Human Genomic Laboratory of the University of Medicine and Pharmacy of Craiova. The analysis was conducted using TaqMan allelic discrimination assays in accordance with the manufacturer’s instructions (Applied Biosystems, Foster City, CA, USA). Genotype calling was performed using the allelic discrimination module of ViiA™ 7 Software version 1.0.

### 4.3. Definitions and Cut-Off Points

#### 4.3.1. Metabolic Risk Clustering

Metabolic syndrome components were defined according to the recently proposed pediatric criteria published by Zong et al. in 2024, which provide clear age- and region-specific cut-off values and allow a standardized assessment of metabolic risk across childhood [18]. These criteria were selected due to their clarity and applicability in pediatric populations, including younger children.

For the assessment of central obesity, waist circumference percentiles (≥90th percentile for age and sex) were used, as this approach remains the most widely validated and commonly applied method in pediatric clinical research. Waist circumference percentiles account for age- and growth-related changes in body proportions throughout childhood and adolescence and are strongly associated with visceral adiposity and cardiometabolic risk.

In addition, the WHtR was also calculated and reported as a complementary anthropometric indicator, in order to facilitate interpretation and comparison with recently published studies using fixed cut-off values. However, waist circumference percentiles were retained as the primary measure of central obesity to ensure methodological consistency within a growth-dependent pediatric cohort.

Finally, MetS components and their corresponding cut-off values were defined as follows:

- Waist circumference (≥90th percentile for age and sex [50]);
- Hypertriglyceridemia:
  ○ Triglycerides ≥ 130 mg/dL in children aged 10–17 years;
  ○ Triglycerides ≥ 100 mg/dL in children aged 6–9 years;
- Low HDL-C: HDL-C < 40 mg/dL;
- Elevated blood pressure:
  ○ Blood pressure ≥ 130/80 mmHg in adolescents aged 13–17 years;
  ○ Blood pressure ≥ 120/80 mmHg in children aged 6–12 years;
- Fasting plasma glucose ≥ 100 mg/dL.

The number of metabolic components derivate from MetS criteria present was calculated for each participant and used as a measure of metabolic risk clustering, rather than as a categorical diagnosis of MetS, as we also included children younger than 10 years of age, for whom there is not a generally accepted definition for MetS. Hence, we presented it not as a present or absent variable, but as a continuous variable ranging from 0 components to 5 components.

#### 4.3.2. Serum Iron and Ferritin

Serum ferritin was considered as a biomarker potentially reflecting both iron metabolism and inflammatory processes. Given its dual role, its interpretation was closely related to serum iron status.

The cutoff points for serum ferritin were in accordance with the values provided by the lab:

- Low levels: ≤14 ng/mL;
- Normal levels: 15–150 ng/mL;
- High levels: ≥151 ng/mL;

Iron deficiency was defined as values below age- and sex-specific reference ranges as follows:

- Under the age of 14 years: 29–137 μg/dL;
- For children aged 14 or above:
  ○ Males: 43–176 μg/dL;
  ○ Females: 33–170 μg/dL.

Based on serum iron and ferritin levels, participants were classified into four categories, to better distinguish between classical iron deficiency and inflammation-related alterations of iron metabolism:

- Normal iron status, characterized by normal serum iron and normal ferritin levels;
- Classical iron deficiency, defined by low serum iron and low ferritin levels;
- Isolated high ferritin, defined by normal serum iron in the presence of increased ferritin levels;
- Discordant iron–ferritin pattern, defined by low serum iron in the presence of increased ferritin levels, suggestive of inflammation-related iron sequestration or altered iron metabolism.

This classification was used to facilitate the adequate interpretation of ferritin levels and to explore their associations with metabolic and hepatic changes.

#### 4.3.3. Hepatic Steatosis and Fibrosis Grades

Liver ultrasonography was performed in B-mode to determine the presence and degree of fat accumulation. Hepatic echogenicity was compared with the right renal cortex. The attenuation of the ultrasound beam with depth-dependent signal loss, blurring of hepatic vascular structures, and reduced visualization of the diaphragm was considered abnormal [53].

To quantify steatosis severity, we used the following categories:

- Mild steatosis: diffuse increased hepatic echogenicity with preserved visualization of periportal and diaphragmatic structures;
- Moderate steatosis: increased echogenicity obscuring periportal details while diaphragmatic visualization remained appreciable;
- Severe steatosis: marked echogenicity with loss of visualization of both periportal and diaphragmatic landmarks;
- Normal liver: the absence of the above-mentioned modifications.

Liver stiffness was assessed using transient elastography with an adapted probe. Ten valid measurements were obtained for each participant, and the mean value (kPa) was recorded. Fibrosis staging was interpreted as follows: 0–6 kPa (F0), 6–7 kPa (F1), and 7–8 kPa (F2). These thresholds were selected based on available literature [54,55]; however, it should be noted that universally validated cut-off values for fibrosis staging in pediatric populations are still lacking.

### 4.4. Statistical Analysis

Data were initially collected and organized using Microsoft^®^ Excel^®^ 2021 MSO (Version 2406, Build 16.0.17726.20078). Statistical analyses were performed using SPSS version 26.0 (SPSS Inc., Chicago, IL, USA). When necessary, English language editing was assisted by ChatGPT (version 4o; OpenAI, San Francisco, CA, USA).

Given the relatively small sample size (n = 68), data distribution was assessed using the Shapiro–Wilk test, which is considered a robust and reliable method for evaluating normality in small to moderate samples. This step was essential for selecting appropriate descriptive statistics and inferential analyses.

The normality assessment revealed that most variables did not follow a normal distribution, a finding that is expected in a heterogeneous pediatric cohort characterized by a wide age range and varying degrees of metabolic and hepatic involvement. Consequently, non-parametric statistical approaches were preferentially applied in subsequent analyses.

According to the Shapiro–Wilk test (*p* > 0.05), the following variables were normally distributed: waist circumference, waist-to-height ratio (WHtR), and glycemia. These variables were therefore expressed as mean ± standard deviation (SD).

In contrast, the following variables showed a non-normal distribution (Shapiro–Wilk *p* < 0.05): age, body mass index (BMI), fibrosis score median, serum iron, serum ferritin, height, systolic blood pressure, diastolic blood pressure, triglycerides, HDL-cholesterol, and the number of metabolic syndrome criteria. These variables were summarized using the median and interquartile range (IQR), defined as the 25th percentile (Q1) and the 75th percentile (Q3).

Categorical variables were presented as absolute frequencies and percentages. Given the predominance of non-normally distributed variables, non-parametric statistical methods were used to explore associations between metabolic, hepatic, and inflammatory parameters.

Given the relatively small sample size, the multivariable analysis was considered exploratory and hypothesis-generating, intending to identify independent associations rather than establish causal or independent predictive relationships. Prior to model construction, potential multicollinearity among predictors was evaluated using variance inflation factors, and no significant collinearity was identified.

All the tables and figures included in the present article were generated and edited using the SPSS software.

## 5. Conclusions

This study showed that ferritin levels were associated with both metabolic risk clustering and hepatic steatosis.

*PNPLA3* G risk allele carriers exhibited a less favorable cardiometabolic profile, while the variant was not independently associated with ferritin levels or fibrosis severity in this cohort.

Ferritin variability in this cohort may reflect the coexistence of metabolic alterations and hepatic alterations, supporting its role as a nonspecific marker rather than a specific mechanistic indicator. The present data do not allow conclusions regarding the relative contribution of inflammatory, metabolic, or genetic mechanisms. In this context, ferritin could represent a simple laboratory parameter that may help identify children with metabolic alterations who require closer clinical evaluation.

Overall, ferritin may represent a nonspecific marker associated with metabolic and hepatic alterations in children; however, its clinical utility and potential prognostic value require confirmation in larger prospective studies including detailed inflammatory profiling.

## Figures and Tables

**Table 1:** Baseline characteristics of the study population.

Variable		Value (n = 68)
Age (years)		10.5 (8.0–12.0)
Gender (n,%)	Males	50 (73.52%)
	Females	18 (26.47%)
Environment (n,%)	Urban	37 (54.41%)
	Rural	31 (45.58%)
BMI (kg/m^2^)		25.65 (19.1–29.3)
Waist circumference ≥ 90th percentile (n,%)		48 (70.6%)
WHtR		0.59 (0.54–0.64)
SBP (mmHg)		113 (108–126.5)
DBP (mmHg)		66 (60.3–80.3)
High SBP/DBP (n,%)		27 (39.7%)
Glycemia (mg/dL)		83.04 ± 11.93
High glycemia (n, %)		4 (5.9%)
Triglycerides (mg/dL)		125.5 (99.0–207.3)
High triglycerides (n,%)		31 (45.6%)
HDL-C, mg/dL (mg/dL)		36.5 (24.6–57.1)
Low HDL-C (n,%)		38 (55.9%)
Serum iron (µg/dL)		11.0 (8.0–18.0)
Serum ferritin (ng/mL)		191.5 (92.3–213.8)
Metabolic risk components	0	19 (27.9%)
	1	5 (7.4%)
	2	9 (13.2%)
	3	17 (25%)
	4	16 (23.5%)
	5	2 (2.9)
Echographic score (hepatic steatosis)	None	19 (27.9%)
	Mild	8 (11.8%)
	Moderate	25 (36.8%)
	Severe	16 (23.5%)
Fibrosis grade	F0	39 (57.4%)
	F1	12 (17.6%)
	F2	17 (25%)
	F3	0
	F4	0
Fibrosis score median (kPa)		5.7 (3.5–7.05)
*PNPLA3*	C/C	28 (41.2%)
	C/G	29 (42.6%)
	G/G	11 (16.2%)

Data are presented as median (interquartile range) or mean ± standard deviation, as appropriate and categorical variables are presented as number (percentage). SBP—Systolic blood pressure; DBP—Diastolic blood pressure; HDL-C—High-density lipoprotein cholesterol; *PNPLA3—Patatin-like phospholipase domain-containing protein 3.*

## Data Availability

The original contributions presented in this study are included in the article. Further inquiries can be directed to the corresponding author.

## Abbreviations

The following abbreviations are used in this manuscript:

BMI	Body mass index
MetS	Metabolic syndrome
MASLD	Metabolic Dysfunction-Associated Steatotic Liver Disease
MAFLD	Metabolic Dysfunction-Associated Fatty Liver Disease
NAFLD	Non-Alcoholic Fatty Liver Disease
T2DM	type 2 Diabetes Mellitus
PNPLA3	Patatin-like phospholipase domain-containing protein 3
WHtR	Waist-to-height ratio
HDL-C	High-density lipoprotein cholesterol
SBP	Systolic blood pressure
DBP	Diastolic blood pressure

## References

[1] Ling J., Chen S., Zahry N.R., Kao T.-S.A. (2023). Economic Burden of Childhood Overweight and Obesity: A Systematic Review and Meta-Analysis. Obes. Rev..

[2] Biazzi Leal D., Altenburg de Assis M.A., Hinnig P.d.F., Schmitt J., Soares Lobo A., Bellisle F., Di Pietro P., Vieira F., De Moura Araujo P.H., De Andrade D. (2017). Changes in Dietary Patterns from Childhood to Adolescence and Associated Body Adiposity Status. Nutrients.

[3] Jebeile H., Kelly A.S., O’Malley G., Baur L.A. (2022). Obesity in Children and Adolescents: Epidemiology, Causes, Assessment, and Management. Lancet Diabetes Endocrinol..

[4] Panganiban J., Kehar M., Ibrahim S.H., Hartmann P., Sood S., Hassan S., Ramirez C.M., Kohli R., Censani M., Mauney E. (2025). Metabolic Dysfunction-Associated Steatotic Liver Disease (MASLD) in Children with Obesity: An Obesity Medicine Association (OMA) and Expert Joint Perspective 2025. Obes. Pillars.

[5] Malecki S.L., Shen T., Loffler A., Stukel T.A., de Oliveira C., Roberts S.B., Bassett A.S., Nelson K.E., Razak F., Verma A.A. (2026). Characteristics and Outcomes of Adults Hospitalized with Childhood-Onset Complex Chronic Conditions. JAMA Netw. Open.

[6] Cura–Esquivel I., Perales-Quintana M.M., Torres-González L., Guzmán-Avilán K., Muñoz-Espinosa L., Cordero-Pérez P. (2023). Metabolic, Inflammatory and Adipokine Differences on Overweight/Obese Children with and without Metabolic Syndrome: A Cross-Sectional Study. PLoS ONE.

[7] Li X., Zhou X.-D., Wu J., Zhao Z., Xie F., Li Y., Li W., Yan X., Sui S., Zhang L. (2025). Pediatric MASLD in China: Epidemiology, Screening, Diagnosis, and Management. Lancet Reg. Health West. Pac..

[8] Miller D.M., McCauley K.F., Dunham-Snary K.J. (2025). Metabolic Dysfunction-Associated Steatotic Liver Disease (MASLD): Mechanisms, Clinical Implications and Therapeutic Advances. Endocrinol. Diabetes Metab..

[9] Moreira A.C., Mesquita G., Gomes M.S. (2020). Ferritin: An Inflammatory Player Keeping Iron at the Core of Pathogen-Host Interactions. Microorganisms.

[10] Fonseca Ó., Ramos A.S., Gomes L.T.S., Gomes M.S., Moreira A.C. (2023). New Perspectives on Circulating Ferritin: Its Role in Health and Disease. Molecules.

[11] Srivastav S.K., Mir I.A., Bansal N., Singh P.K., Kumari R., Deshmukh A. (2022). Serum Ferritin in Metabolic Syndrome—Mechanisms and Clinical Applications. Pathophysiology.

[12] Suárez-Ortegón M.F., Blanco E., McLachlan S., Fernandez-Real J.M., Burrows R., Wild S.H., Lozoff B., Gahagan S. (2019). Ferritin Levels throughout Childhood and Metabolic Syndrome in Adolescent Stage. Nutr. Metab. Cardiovasc. Dis..

[13] Stepan M.D., Vintilescu Ș.B., Ionele C.M., Dumitra G.G., Podeanu M.A., Bigea C.C., Sacerdoțianu V.M., Anastasescu C.M., Florescu D.N. (2024). Associations of Ultrasound Findings with Serum Iron and Ferritin Levels in Children with Obesity. Life.

[14] Abaturov A., Nikulina A. (2023). Role of Genetic Modification of the PNPLA3 Gene in Predicting Metabolically Unhealthy Obesity and Metabolic Associated Fatty Liver Disease in Children. Eur. J. Clin. Exp. Med..

[15] Browning J.D., Cohen J.C., Hobbs H.H. (2010). Patatin-Like Phospholipase Domain-Containing 3 and the Pathogenesis and Progression of Pediatric Nonalcoholic Fatty Liver Disease. Hepatology.

[16] Valentini D., Mosca A., Di Camillo C., Crudele A., Sartorelli M.R., Scoppola V., Tarani L., Villani A., Raponi M., Novelli A. (2020). PNPLA3 Gene Polymorphism Is Associated with Liver Steatosis in Children with Down Syndrome. Nutr. Metab. Cardiovasc. Dis..

[17] Mohammadi M., Gozashti M.H., Aghadavood M., Mehdizadeh M.R., Hayatbakhsh M.M. (2017). Clinical Significance of Serum IL-6 and TNF-α Levels in Patients with Metabolic Syndrome. Rep. Biochem. Mol. Biol..

[18] Zong X., Kelishadi R., Kim H.S., Schwandt P., Matsha T.E., Mill J.G., Caserta C.A., Medeiros C.C.M., Kollias A., Whincup P.H. (2024). A Proposed Simplified Definition of Metabolic Syndrome in Children and Adolescents: A Global Perspective. BMC Med..

[19] Hampl S.E., Hassink S.G., Skinner A.C., Armstrong S.C., Barlow S.E., Bolling C.F., Edwards K.C.A., Eneli I., Hamre R., Joseph M.M. (2023). Clinical Practice Guideline for the Evaluation and Treatment of Children and Adolescents with Obesity. Pediatrics.

[20] Codazzi V., Frontino G., Galimberti L., Giustina A., Petrelli A. (2024). Mechanisms and Risk Factors of Metabolic Syndrome in Children and Adolescents. Endocrine.

[21] Stepan M.D., Vintilescu Ș.B., Streață I., Podeanu M.A., Florescu D.N. (2023). The Role of Vitamin D in Obese Children with Non-Alcoholic Fatty Liver Disease and Associated Metabolic Syndrome. Nutrients.

[22] Talebi Anaraki K., Heidari-Beni M., Arefian M., Kelishadi R. (2025). Managing Pediatric Metabolic Syndrome: A Systematic Review of Current Approaches. BMC Pediatr..

[23] de Lamas C., Kalén A., Anguita-Ruiz A., Pérez-Ferreirós A., Picáns-Leis R., Flores K., Moreno L.A., Bueno G., Gil Á., Gil-Campos M. (2022). Progression of Metabolic Syndrome and Associated Cardiometabolic Risk Factors from Prepuberty to Puberty in Children: The PUBMEP Study. Front. Endocrinol..

[24] Jia S., Ye X., Wu T., Wang Z., Wu J. (2025). Global Prevalence of Metabolic Dysfunction-Associated Fatty Liver Disease in Children and Adolescents with Overweight and Obesity: A Systematic Review and Meta-Analysis. BMC Gastroenterol..

[25] Ciardullo S., Monti T., Perseghin G. (2021). Prevalence of Liver Steatosis and Fibrosis Detected by Transient Elastography in Adolescents in the 2017–2018 National Health and Nutrition Examination Survey. Clin. Gastroenterol. Hepatol..

[26] Fajrudheen M., Mahapatro S., Panigrahi M.K., Naik S., Satapathy A.K. (2025). Noninvasive Assessment of Nonalcoholic Fatty Liver Disease in Children with Overweight and Obesity by Transient Elastography. Indian J. Endocrinol. Metab..

[27] Jin H.Y., Noh E.S., Jeong H., Hwang I.T. (2024). Prediction of Hepatic Fibrosis Using the Aspartate Transaminase-to-Platelet Ratio Index in Children and Adolescents with Metabolic Dysfunction-Associated Steatotic Liver Disease. BMC Pediatr..

[28] Karamfilova V., Gateva A., Assyov Y., Alexiev A., Savov A., Yaneva N., Ivanova I., Ivanova-Boyanova R., Ivanova R., Vlahova Z. (2019). PNPLA3 I148M Polymorphism in Patients with Nonalcoholic Fatty Liver Disease, Obesity and Prediabetes. J. Gastrointest. Liver Dis..

[29] Stasinou E., Emmanouilidou-Fotoulaki E., Kavga M., Sotiriadou F., Lambropoulos A.F., Fotoulaki M., Papadopoulou-Legbelou K. (2022). Association of Rs738409 Polymorphism in Adiponutrin Gene with Liver Steatosis and Atherosclerosis Risk Factors in Greek Children and Adolescents. Nutrients.

[30] Tang S., Zhang J., Mei T.T., Guo H.Q., Wei X.H., Zhang W.Y., Liu Y.L., Liang S., Fan Z.P., Ma L.X. (2020). Association of PNPLA3 Rs738409 G/C Gene Polymorphism with Nonalcoholic Fatty Liver Disease in Children: A Meta-Analysis. BMC Med. Genet..

[31] Santoro N., Kursawe R., D’Adamo E., Dykas D.J., Zhang C.K., Bale A.E., Calí A.M., Narayan D., Shaw M.M., Pierpont B. (2010). A Common Variant in the Patatin-Like Phospholipase 3 Gene (PNPLA3) Is Associated with Fatty Liver Disease in Obese Children and Adolescents. Hepatology.

[32] Targher G., Mantovani A., Alisi A., Mosca A., Panera N., Byrne C.D., Nobili V. (2019). Relationship Between PNPLA3 Rs738409 Polymorphism and Decreased Kidney Function in Children With NAFLD. Hepatology.

[33] Sun D.Q., Zheng K.I., Xu G., Ma H.L., Zhang H.Y., Pan X.Y., Zhu P.W., Wang X.D., Targher G., Byrne C.D. (2020). PNPLA3 Rs738409 Is Associated with Renal Glomerular and Tubular Injury in NAFLD Patients with Persistently Normal ALT Levels. Liver Int..

[34] Goran M.I., Walker R., Le K.A., Mahurkar S., Vikman S., Davis J.N., Spruijt-Metz D., Weigensberg M.J., Allayee H. (2010). Effects of PNPLA3 on Liver Fat and Metabolic Profile in Hispanic Children and Adolescents. Diabetes.

[35] Xia M.F., Ling Y., Bian H., Lin H.D., Yan H.M., Chang X.X., Li X.M., Ma H., Wang D., Zhang L.S. (2016). I148M Variant of PNPLA3 Increases the Susceptibility to Non-Alcoholic Fatty Liver Disease Caused by Obesity and Metabolic Disorders. Aliment. Pharmacol. Ther..

[36] Dongiovanni P., Donati B., Fares R., Lombardi R., Mancina R.M., Romeo S., Valenti L. (2013). PNPLA3 I148M Polymorphism and Progressive Liver Disease. World J. Gastroenterol..

[37] Ko J.S. (2019). New Perspectives in Pediatric Nonalcoholic Fatty Liver Disease: Epidemiology, Genetics, Diagnosis, and Natural History. Pediatr. Gastroenterol. Hepatol. Nutr..

[38] Goyal N.P., Schwimmer J.B. (2017). The Genetics of Pediatric Nonalcoholic Fatty Liver Disease (NAFLD). Clin. Liver Dis..

[39] Astarini F.D., Ratnasari N., Wasityastuti W. (2022). Update on Non-Alcoholic Fatty Liver Disease-Associated Single Nucleotide Polymorphisms and Their Involvement in Liver Steatosis, Inflammation, and Fibrosis: A Narrative Review. Iran. Biomed. J..

[40] Fruntelată R.F., Bakri A., Stoica G.A., Mogoantă L., Ionovici N., Popescu G., Vasilica Pîrşcoveanu D.F., Raicea A., Ciurea M.E. (2023). Assessment of Tumoral and Peritumoral Inflammatory Reaction in Cutaneous Malignant Melanomas. Rom. J. Morphol. Embryol..

[41] Podeanu M.-A., Vintilescu Ș.B., Sandu R.E., Ionele C.M., Niculescu C.E., Florescu M.-M., Șelaru E.-L., Stepan M.D. (2025). Ferritin as an Inflammatory Marker in Pediatric Metabolic Syndrome: Links to Obesity and Liver Ultrasound Alterations. Int. J. Mol. Sci..

[42] Crasan I.-M., Tanase M., Delia C.E., Gradisteanu-Pircalabioru G., Cimpean A., Ionica E. (2025). Metaflammation’s Role in Systemic Dysfunction in Obesity: A Comprehensive Review. Int. J. Mol. Sci..

[43] Masenga S.K., Kabwe L.S., Chakulya M., Kirabo A. (2023). Mechanisms of Oxidative Stress in Metabolic Syndrome. Int. J. Mol. Sci..

[44] Suárez-Ortegón M.F., Ensaldo-Carrasco E., Shi T., McLachlan S., Fernández-Real J.M., Wild S.H. (2018). Ferritin, Metabolic Syndrome and Its Components: A Systematic Review and Meta-Analysis. Atherosclerosis.

[45] Abril-Ulloa V., Flores-Mateo G., Solà-Alberich R., Manuel-y-Keenoy B., Arija V. (2014). Ferritin Levels and Risk of Metabolic Syndrome: Meta-Analysis of Observational Studies. BMC Public Health.

[46] Zhang H., Wang L., Li S., Liu X., Li Y., He Y., Man Q., Yang L. (2020). Association of Iron Storage Markers with Metabolic Syndrome and Its Components in Chinese Rural 6–12 Years Old Children: The 2010–2012 China National Nutrition and Health Survey. Nutrients.

[47] Podeanu M.-A., Ionele C.M., Sandu R.E., Rogoveanu I., Stepan M.D., Niculescu C.E., Cazacu S.-M., Vintilescu Ș.B. (2026). Obesity, Metabolic Syndrome and MASLD in Children: Inflammation as the Missing Link—A Short Narrative Review. Life.

[48] Zhang J., Cao J., Xu H., Dong G., Huang K., Wu W., Ye J., Fu J. (2021). Ferritin as a Key Risk Factor for Nonalcoholic Fatty Liver Disease in Children with Obesity. J. Clin. Lab. Anal..

[49] Valenti L. (2012). Diagnostic and Therapeutic Implications of the Association between Ferritin Level and Severity of Nonalcoholic Fatty Liver Disease. World J. Gastroenterol..

[50] Baker J.L., Farpour-Lambert N.J., Nowicka P., Pietrobelli A., Weiss R. (2010). Evaluation of the Overweight/Obese Child—Practical Tips for the Primary Health Care Provider: Recommendations from the Childhood Obesity Task Force of the European Association for the Study of Obesity. Obes. Facts.

[51] MSD Manuals Professional Version. Blood Pressure Percentiles for Boys (2–17 Years). https://www.msdmanuals.com/professional/multimedia/clinical-calculator/blood-pressure-percentiles-for-boys-2-17-years.

[52] MSD Manuals Professional Version. Blood Pressure Percentiles for Girls (2–17 Years). https://www.msdmanuals.com/professional/multimedia/clinical-calculator/blood-pressure-percentiles-for-girls-2-17-years.

[53] Di Martino M., Koryukova K., Bezzi M., Catalano C. (2017). Imaging Features of Non-Alcoholic Fatty Liver Disease in Children and Adolescents. Children.

[54] Zeng J., Zhang X., Sun C., Pan Q., Lu W.-Y., Chen Q., Huang L.-S., Fan J.-G. (2019). Feasibility Study and Reference Values of FibroScan 502 with M Probe in Healthy Preschool Children Aged 5 Years. BMC Pediatr..

[55] Jain V., Poddar U., Negi T.S., Saraswat V.A., Krishnani N., Yachha S.K., Srivastava A. (2020). Utility and Accuracy of Transient Elastography in Determining Liver Fibrosis: A Case-Control Study. Eur. J. Pediatr..

